# Genetic associations with mathematics tracking and persistence in secondary school

**DOI:** 10.1038/s41539-020-0060-2

**Published:** 2020-02-05

**Authors:** K. Paige Harden, Benjamin W. Domingue, Daniel W. Belsky, Jason D. Boardman, Robert Crosnoe, Margherita Malanchini, Michel Nivard, Elliot M. Tucker-Drob, Kathleen Mullan Harris

**Affiliations:** 1 Department of Psychology and Population Research Center, University of Texas at Austin, Austin, TX USA; 2 Graduate School of Education, Stanford University, Stanford, CA USA; 3 Department of Epidemiology, Columbia University Mailman School of Public Health, New York, NY USA; 4 Department of Sociology and Institute of Behavioral Science, University of Colorado at Boulder, Boulder, CA USA; 5 Department of Sociology and Population Research Center, University of Texas at Austin, Austin, TX USA; 6 Biological Psychology, VU University Amsterdam, Amsterdam, The Netherlands; 7 Department of Sociology and Carolina Population Center, University of North Carolina at Chapel Hill, Chapel Hill, NC USA

**Keywords:** Human behaviour, Education

## Abstract

Maximizing the flow of students through the science, technology, engineering, and math (STEM) pipeline is important to promoting human capital development and reducing economic inequality. A critical juncture in the STEM pipeline is the highly cumulative sequence of secondary school math courses. Students from disadvantaged schools are less likely to complete advanced math courses. Here, we conduct an analysis of how the math pipeline differs across schools using student *polygenic scores*, which are DNA-based indicators of propensity to succeed in education. We integrated genetic and official school transcript data from over 3000 European-ancestry students from U.S. high schools. We used polygenic scores as a molecular tracer to understand how the flow of students through the high school math pipeline differs in socioeconomically advantaged versus disadvantaged schools. Students with higher education polygenic scores were tracked to more advanced math already at the beginning of high school and persisted in math for more years. Analyses using genetics as a molecular tracer revealed that the dynamics of the math pipeline differed by school advantage. Compared to disadvantaged schools, advantaged schools buffered students with low polygenic scores from dropping out of math. Across all schools, even students with exceptional polygenic scores (top 2%) were unlikely to take the most advanced math classes, suggesting substantial room for improvement in the development of potential STEM talent. These results link new molecular genetic discoveries to a common target of educational-policy reforms.

## Introduction

Math matters for economic success.^1^ American students who take math courses beyond Algebra 2 are more likely to enroll in college and complete a STEM degree^2–4^ and have better labor market outcomes.^5–7^ Students from low-income families and schools are less likely to take advanced math courses in secondary school, which impairs their entry to post-secondary STEM education and ultimately to a STEM career.^8–10^ There are, however, continuing debates about whether the underrepresentation of low-income students in STEM is due to the diminished resources available to their schools and families or, rather, due to those students having lower aptitude or interest in math.^8,11–14^ Despite the intense focus on STEM outcomes, it is challenging to conduct rigorous studies of whether and how schools differ in the flow of students through the math pipeline. In particular, analyses that statistically control for traditional measures of student aptitude or interest might lead to biased conclusions about how the math pipeline differs across schools, because these student characteristics can themselves be influenced by previous educational experiences.^8^

Our project addresses the challenge of understanding how students’ progress through the STEM pipeline might vary as a function of school characteristics by using a DNA-based measure of students’ likelihood to succeed in education. A previous genome-wide association study (GWAS) of 1.1 million people identified hundreds of genetic variants associated with higher educational attainment.^15^ These results can be used to calculate an *education polygenic score* (education-PGS), which is a composite index of genetic variants associated with completing more years of school.^16–18^ The education-PGS predicts whether or not an individual completes college about as well as his/her family income does.^15^ Moreover, unlike traditional measures of student aptitude, individual differences in genetic sequence are fixed at conception and cannot be changed by educational experiences.

Polygenic scores can therefore be used as a molecular tracer to measure flows of students through the STEM pipeline and assess how these flows differ across schools. Just as a radiologist might administer a radioactive tracer to track the flow of blood within the body, researchers can use genetics as a molecular tracer to get a clearer image of how students progress through the twists and turns of the educational system. Here, we use polygenic scores to follow the curricular histories of students who attended secondary schools with varying levels of socioeconomic advantage. This approach offers a way of diagnosing the extent to which students who have high genetic propensities for success in education leak out of the STEM pipeline by failing to advance in their mathematics training.

In mapping the flow of students through the secondary school math curriculum, we focus on two dimensions of high school mathematics coursetaking—*tracking* and *persistence*. In some countries (e.g., Germany), students are tracked into different types of secondary schools at a discrete number of branch points. The U.S., in contrast, does not have a formal tracking system. Instead, students are offered curricular options that are differentiated by content and difficulty (e.g., Pre-Algebra vs. Algebra I vs. Algebra II). Students are informally *tracked* toward final math credentials via their course placement in the first year of secondary school (or earlier).^19^ As subsequent coursetaking hinges on successful completion of pre-requisites and mastery of cumulative content, students’ curricular decisions become strongly path-dependent.^19–21^ Students additionally vary after the first year in whether they *persist* in their track throughout secondary school, move to a less-advanced track, or discontinue mathematics training entirely.

We first sought to validate the education-PGS as a molecular tracer by testing whether it predicted being tracked into a more advanced math class at the beginning of high school and whether it predicted persisting in math for longer. Next, we used the education-PGS to examine differences between schools in the flow of students through the STEM pipeline. Specifically, we focused on the difference between schools that served mainly students from well-educated families versus schools that served mainly students from families with less formal education. We found that the education-PGS predicts movement through the math coursetaking pipeline but that it also intersects with school characteristics. We are able to use our genetic analysis to make an important observation about the role of schools independent of genetic variation. In particular, we observe that two students with the *same* education-PGS might differ substantially in their progress through the STEM pipeline, depending on their school characteristics.

While our approach leverages insights from large-scale genetic studies, we emphasize that genetics are clearly not the only factor that matters for student achievement. Indeed, by comparing students who are equivalent in their measured genetic propensities, but who attend different schools, our analyses can illuminate how mathematics achievement depends on contextual factors. In this way, we view research that uses genetic tools as complementing educational research on how school-based factors, such as instructional practices, can boost mathematics achievement.^22^

## Results

### Mathematics coursetaking can be categorized from school transcripts

Analyses used genetic and official school transcript data on *N* = 3,635 unrelated adolescents from the National Longitudinal Study of Adolescent to Adult Health (Add Health, see Methods; Supplementary Fig. 1).^23^ Respondents were enrolled in a U.S. high school in 1994–1995. We restricted analyses to European-ancestry participants to prevent inadvertently conflating genetic variation with racial or ethnic background. Previous analyses of national population patterns have revealed a fairly standardized sequence of math coursework, ranging from more basic courses like Pre-Algebra to more advanced courses like Calculus.^24,25^ We used this sequence to categorize each participant’s math coursework across four years of secondary school, based on information obtained from schools, including course catalogs, school information forms, and interviews with school administrators (Supplementary Table 2).

At the beginning of secondary school (9th grade, age ~14 years), most students were enrolled in Algebra 1 (51%), but some students were tracked to less advanced (Pre-Algebra or below, 29%) or more advanced (Geometry or above, 20%) courses. A student’s final level of mathematics training was strongly dependent on 9th-grade course enrollment: 44% of those enrolled in Geometry or higher in 9th-grade ultimately completed Calculus, compared to only 4.2% of those enrolled in Algebra 1 and 1% enrolled in Pre-Algebra or lower level math class.

### Student polygenic score predicts mathematics tracking

**Fig. 1:**
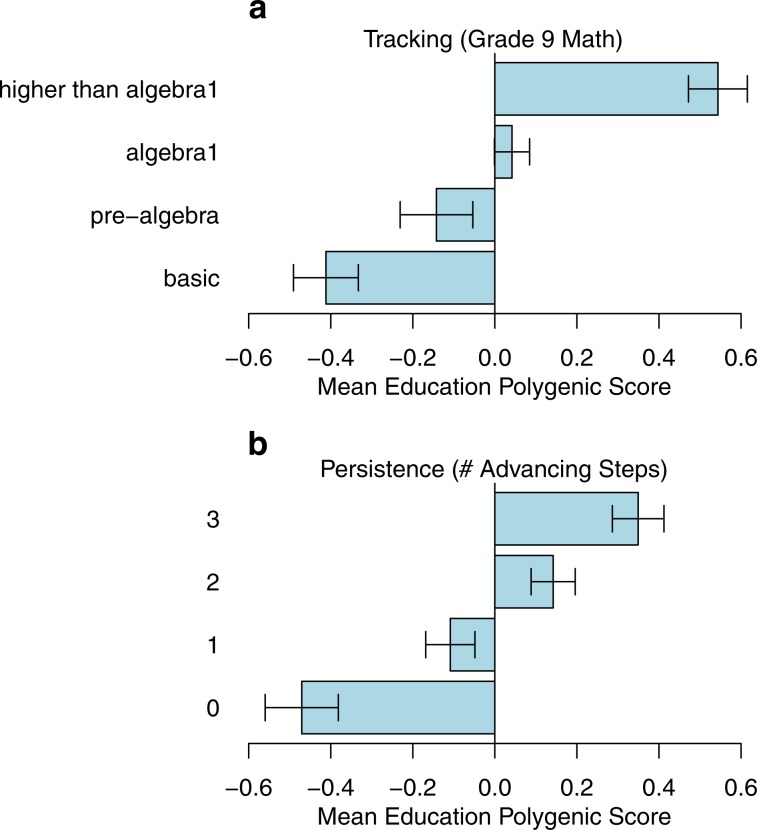
Students with higher education-associated polygenic scores are tracked to more advanced math and persist for longer in math. Error bars represent 95% confidence intervals around the mean.

Students with higher polygenic scores were more likely to be tracked into more advanced math courses in 9th grade (Fig. 1a, *b* = 0.583, SE = 0.035, 95% CI *=* [0.516, 0.656], Supplementary Table 3). However, Add Health participants with a higher education-PGS more often grew up in high-SES families and attended high-SES schools, as compared to participants with lower polygenic scores.^26,27^ These gene-environment correlations raise the possibility that genetic associations with mathematics tracking could be due to clustering of students with higher polygenic scores in environmental contexts that better support math achievement. To address this possibility, we repeated our analysis of tracking in the 9th grade using measures of school-SES and family SES as covariates (Methods, Supplementary Table 3). As expected, students from higher SES families were tracked to more advanced math courses at the beginning of secondary school (*b* = 0.419, SE = 0.039, 95% CI *=* [0.344, 0.5]). The association between school-SES and tracking was also positive but not significantly different than zero (*b* = 0.704, SE = 0.571, 95% CI *=* [−0.671, 1.74]). However, including family SES and school-SES as covariates attenuated the association between the education-PGS and mathematics tracking in the 9th-grade only by about 20% (attenuated from *b* = 0.583, SE = 0.035, to *b* = 0.469, SE = 0.035, 95% CI *=* [0.397, 0.53], Supplementary Table 3). Note that the association with genetics was roughly comparable in magnitude to the association with family SES. As a stronger test of whether the genetic association with mathematics tracking was due to clustering of students with high education-PGS into certain schools, we repeated our analysis of 9th-grade tracking yet again, this time using school-fixed-effects regression to compare students to their schoolmates (Supplementary Table 3).^28^ Comparing only students who were in Algebra 1 or below, students with higher education-PGS were less likely, compared to their schoolmates, to be placed in a remedial track (Pre-Algebra or lower) than in Algebra 1 (*b* = 0.387, SE = 0.054, 95% CI *=* [0.294, 0.504]). Similarly, comparing only students who were in Algebra 1 or above, students with higher education-PGS were more likely, compared to their schoolmates, to be placed in an advanced track (Geometry or higher) rather than in Algebra 1 (*b* = 0.587, SE = 0.047, 95% CI *=* [0.501, 0.681]).

### Student polygenic score predicts persistence in mathematics coursetaking

What happens to students after the 9th-grade? Participants in this sample attended high school in the mid-1990 s, when the average high school graduation requirement in U.S. states was 2.4 years of math coursework.^29^ Rates of math drop-out accelerated in later years of secondary school (9th-grade: 2.6%, 10th-grade: 5.2%, 11th-grade: 17.6%, 12th-grade: 44.7%; Supplementary Table 2). Once students dropped out of math, they tended to remain out of math coursework; only 8% of students enrolled in a math class after a year of no math. We summarized persistence across the four years of transcript follow-up as number of advancing steps in the math coursework sequence, ranging from zero to three. For example, a student who completed Algebra 1, Geometry, and Algebra 2 in the first three years of secondary school but who did not take a math course in the 12th-grade, took two advancing steps.

Students with higher education-PGS took more advancing steps (Fig. 1b; *b* = 0.139, SE = 0.011, 95% CI *=* [0.117, 0.159]; Supplementary Table 4). We then repeated this analysis using a number of additional covariates. As we observed for tracking, students from higher SES families were more likely to persist in math coursetaking across secondary school (*b* = 0.120, SE = 0.015, 95% CI *=* [0.089, 0.147]). The association with school-SES was similarly positive but not statistically significant (*b* = 0.234, SE = 0.0171, 95% CI *=* [−0.098, 0.572]). But the education-PGS association with persistence was only modestly attenuated after accounting for family- and school-SES covariates (*b* = 0.096, SE = 0.013, 95% CI *=* [0.072, 0.12]). We also considered a model including 9th-grade course placement as a covariate. Students who were tracked to Pre-Algebra or lower in the 9th-grade persisted less in math than those in Algebra 1 (*b* = −0.221, SE = 0.038, 95% CI *=* [−0.293, −0.147]). In contrast, students in more advanced math tracks in 9th-grade (Geometry or higher) did not differ from those placed in Algebra 1 (*b* = −0.059, SE = 0.044, 95% CI *=* [−0.147, 0.020]). Controlling for tracking in the 9th-grade, the education-PGS again remained associated with persistence (*b* = 0.087, SE = 0.012, 95% CI *=* [0.061, 0.11]). While these analyses suggest a robust association between the educational attainment PGS and persistence, within-family analyses suggested that the polygenic score is not predictive of the sibling with a higher PGS being more persistent than the other (see SI) although this finding could partially be a function of that sample being of limited size.

As with tracking, we repeated this analysis yet again using school fixed-effects to compare students to others in their school. Consistent with previous analyses, participants with higher education-PGSs took more advancing steps in their mathematics coursetaking than their schoolmates (*b* = 0.117, SE = 0.013, 95% CI *=* [0.091, 0.138]; Supplementary Table 4).

**Fig. 2:**
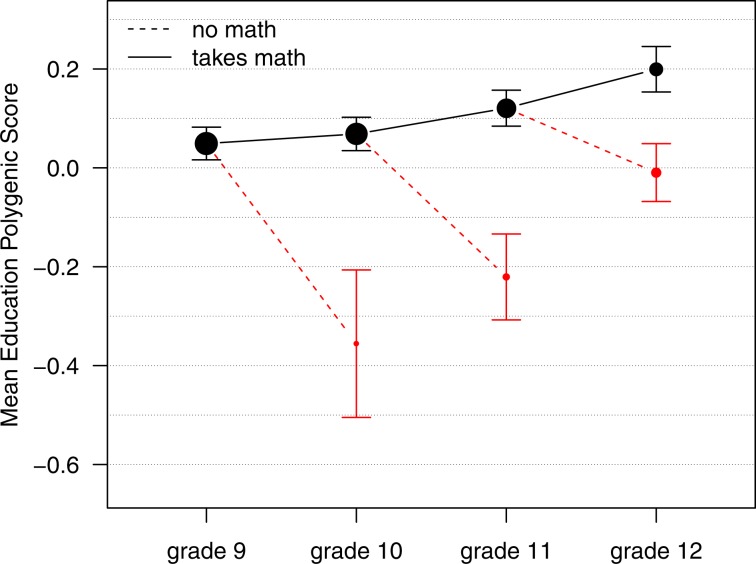
Genetic associations with persistence in math recur year-after-year. Error bars represent 95% confidence intervals around the mean. Size of the dots represents number of students enrolled or not enrolled in math in each year.

We next analyzed persistence on a year-by-year basis. As shown in Fig. 2, most students were enrolled in math in 9th-grade, and their mean education-PGS was the sample mean (i.e., zero). Few of these students dropped out of math in 10th-grade, but these early drop-outs had a low average education-PGS (less than 0.3 SD below the mean). The pace of attrition increased in subsequent years (note growth in size of the red dots), and students who continued to take any math class were an increasingly positively selected group.

We considered whether the education-PGS provided any novel information above and beyond what could be observed from students’ performance in math class. It did. This set of analyses focused on students who were enrolled in any math class in the 9th-, 10th-, and 11th-grades, and tested enrollment in any math class in the subsequent year. End-of-year grade point averages (GPAs; on a 4-point scale) in math were obtained from the school transcripts. At every year, students from higher SES families, students attending higher SES schools, and students who had higher math GPAs were more likely to enroll in math the subsequent year (Supplementary Table 5). After controlling for these covariates, a 1-SD increase in the education-PGS was still associated with 1.26 times greater odds of taking a math class in 10th-grade (95% CI = [1.05–1.56]), 1.15 times greater odds in 11th-grade (95% CI = [1.08–1.28]), and 1.13 times greater odds in the 12th-grade (95% CI = [1.05–1.22]).

**Fig. 3:**
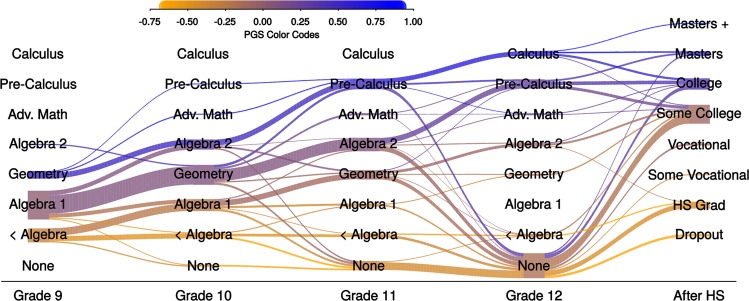
Student DNA can be used to visualize the flow of students through the high school math curriculum. Columns represent year of secondary school; rows represent mathematics course sequence ranging from least to most advanced. Width of the rivers connecting columns proportional to number of students. Shading of rivers represents the average education polygenic score for students in a particular course in a particular year, ranging from low (orange) to high (blue).

Our analyses reveal genetically stratified flows of students through the mathematics training pipeline. We visualized these flows using a “river plot” (Fig. 3).^30^ In the river plot, participants’ math courses (rows) are plotted by year of secondary education (columns). Courses are ordered from most advanced at the top of the graph to least-advanced at the bottom. The widths of the rivers (i.e., the edges connecting row-column nodes) indicate the number of students moving from one course to another. The color of the rivers represents the average education-PGS for students following a particular path (higher in blue, lower in orange). Collectively, these results support the premise that the education-PGS can be used as a molecular tracer to evaluate how students flow through the STEM pipeline in secondary school.

### Higher SES schools buffer the risks faced by students predicted to struggle in math

**Fig. 4:**
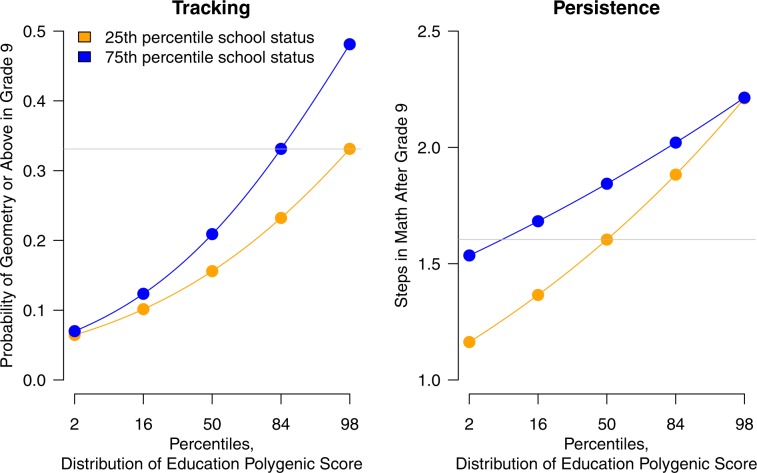
Student tracking and persistence vary as a function of school. **a** Students with high education-associated polygenic scores are more likely to be tracked into advanced math in advantaged schools than in disadvantaged schools. Fitted probabilities of being tracked to Geometry or higher in the 9th grade, based on cumulative link logit model. School status measured by percent of students whose mother graduated from high school. See Supplementary Table 3 for full model results. **b** Students with low education-associated polygenic scores persist more in math in advantaged schools than in disadvantaged schools. Model-implied number of advancing steps from 9–to–12th-grade, based on Poisson model. At least 1 year of math beyond the 9th grade was compulsory in most U.S. states. See Supplementary Table 4 for full model results.

Building on recent evidence,^26,31^ we next conducted two analyses of how STEM pipeline dynamics varied by school advantage. First, we tested if the genetic association with tracking differed between high- and low-SES schools using cumulative link models with product terms to capture interactions between school-SES and the education PGS (Fig. 4a). The interaction term was positive, suggesting that the education-PGS predicted 9th-grade tracking more strongly among students in higher-status schools than in lower-status schools, but this effect was not statistically significant (interaction *b* = 0.59, SE = 0.291, 95% CI [−0.007, 1.11]); Supplementary Table 3). A student with an education-PGS of + 1 (top 16th percentile) who is in a high-status school has a 33.1% probability of being tracked to Geometry in the 9th-grade (note horizontal gray line in Fig. 4a). In order to have the same probability of being placed in Geometry, a student in a low-status school would need to have an education-PGS of + 2.0 (top 2%). Robustness analyses using non-parametric LOESS and adjacent-category logit models suggested similar patterns (see Supplementary Fig. 2).

Second, we tested the interaction between education-PGS and school-SES in predicting number of advancing steps in math. There was a significant and negative interaction on mathematics persistence, such that low-PGS students were *less* likely to drop out of math if attending high-status schools as compared to low-status schools (*b* = −0.304, SE = 0.074, 95% CI *=* [−0.443, −0.147]; Supplementary Table 4). The interaction effect was similar when including 9th-grade tracking as a covariate (*b* = −0.282, SE = 0.072, 95% CI *=* [−0.41, −0.147]). Figure 4b shows how the number of advancing steps varied as a function of education-PGS in schools at the 0.25 quantile versus 0.75 quantile of school status. High-PGS students persisted about equally in their mathematics training regardless of school status. In contrast, students with an average or low education-PGS were particularly likely to drop out of math in low-SES schools. For example, students attending low-SES schools with an average education-PGS completed 1.6 advancing steps (note gray horizontal line in Fig. 4b). However, in high-SES schools, a similarly low level of mathematics persistence is only seen in students at the very low end of the genetic distribution (education-PGS = −1.5, bottom 7%-ile).

Our final analyses focused on school differences in whether or not a student completed Calculus, the most advanced course category in the 9-course sequence. Results from a logistic regression found that school-SES and the education-PGS each predicted taking Calculus, but they did not significantly interact (Supplementary Table 6). Students with an average education-PGS had nearly twice the chances of taking Calculus in a high-SES school (11%) than in a low-SES one (6%). Calculus was rare even among students with exceptional polygenic scores (top 2%, or +2 SD above the mean): High-PGS students had a 24% probability of taking Calculus in a low-SES school and a 31% probability in a high-SES school.

## Discussion

We used data on student genetics as a molecular tracer to test how the flow of students through the high school math curriculum varied between disadvantaged versus advantaged school contexts. Students with higher education polygenic scores tended to enroll in more advanced mathematics tracks in the 9th-grade, and they were more likely to persist in these tracks through the end of high school. Furthermore, genetic associations with tracking and persistence were not explained by differences between schools or measured differences in family socioeconomic status (SES).

However, these student-level results might be complicated by the existence of gene-environment interactions, as the flow of students through the mathematics pipeline differed between high-SES and low-SES schools. Students with low polygenic scores were buffered from dropping out of math in high-status schools. Consequently, students in high-status schools had substantially better math credentials by the end of high school, compared to students who had comparable polygenic scores but who were enrolled in low-status schools. This study cannot identify specific causal factors for school-level differences. Such factors could include school resources and instructional practices (e.g.,^22^), as well as correlated features of neighborhoods and other environmental contexts. Nevertheless, whatever the cause of these school-level differences, our results underscore that many students are not going as far in mathematics as we might expect were they at another school. Furthermore, our results contrast with previous suggestions that school differences in academic outcomes might solely reflect differences in the genetic composition of their student bodies.^13^

Our findings suggest that genetics may provide a novel approach to studying challenging educational problems. A persistent methodological problem in educational research is how to separate out the effects of teachers and schools from the effects of student characteristics that are non-randomly distributed across schools, such as family income.^32^ Observable student characteristics are in flux during these crucial years of development and are also associated with both upstream and downstream developmental influences, such as previous schooling. Genetics, as a fixed characteristic of the student that is as predictive of success in schooling as family income,^15^ offers researchers an additional tool for studying how student development varies by context.

With the caveat that the Add Health data represents an earlier cohort of students, our results further suggest that even advantaged school contexts do a poor job of maximizing human capital. Out of students who both had exceptional polygenic scores (+2SD above the Add Health sample mean or the top 2% of the distribution) and attended high status high school, about 31% took Calculus by the end of high school, whereas only 24% of students who had the same score and attended low-status schools took calculus. This deficit in advanced coursetaking among students with exceptional genetic propensities for succeeding in education suggests that many students who likely could excel in mathematics are not being put in opportunities to do so. In terms of our running metaphor: the pipeline is leaking. Badly.

We acknowledge several limitations. First, these analyses do not inform our understanding of disparities between ethnic and racial groups, which is one of the most pressing problems in the U.S. educational system.^33^ Polygenic scores are useful only for understanding individual differences between people who share the same genetic ancestry, and the validity of education GWAS results has been established only for people of European ancestry.^15^ The extent to which results will generalize to other populations is uncertain. Just as the biomedical use of polygenic scores developed in European-ancestry populations has the potential to exacerbate pre-existing health disparities, using polygenic scores in educational contexts also has the potential to exacerbate pre-existing achievement gaps between racial and ethnic groups.^34^

Second, the genetic predictor deployed here captures only a fraction of the genotypic variation relevant for education. Associations with the polygenic scores are attenuated by measurement error,^35^ and other variants (e.g., rare variants^36^) relevant for ultimate educational attainment might not operate through the processes described here. We anticipate that genetic associations with mathematics coursetaking and other academic achievement outcomes will increase in magnitude as the sample sizes of GWASs continue to increase and as polygenic scores incorporate information from whole-genome sequencing.^37^

Third, our analyses lacked information on specific features of policy or programming that might explain why students with similar genetics fare differently in different schools. Our results suggest that the relevant factors are linked in some way to the socioeconomic characteristics of the students served. Future research is needed to identify what modifiable features of students’ educational environments are involved in amplifying or suppressing genetic influences on their skill development as they move through the STEM pipeline. We encourage researchers to investigate the panoply of institutional, social, demographic, and cultural differences that exist between schools and that might contribute to school-level differences in the association between student genetics and academic achievement.

Fourth, previous studies have suggested that up to half of the polygenic score association with educational outcomes might operate indirectly. In addition to giving information about his/her own biology, a student’s polygenic score also reflects the genotype of his/her parents, which is in turn associated with the environment that the parents provide.^38^ Consequently, the association between genotype and math curricular choices might partially operate not through the genetically-influenced characteristics of the student herself, but through the genetically-influenced characteristics of her parents, such as the greater knowledge that college-educated parents have about how to navigate a differentiated curriculum.^39^ Work is already beginning to document such pathways.^40–42^ Disentangling such indirect genetic effects^43^ from genetic effects that operate through the biology of the student will require larger samples of genetic relatives, such as parent-offspring trios.^44^ We conducted an initial exploration of this question by comparing siblings raised in the same family (Supplement), but the relatively small number of sibling pairs with transcript data available in Add Health limits the definitiveness of our conclusions about the role of indirect genetic effects.

Fifth, there is limited information on the educational histories of students prior to the 9th-grade, but students’ secondary school experiences are, of course, shaped by their previous mathematics skill development and curricular choices. We suspect that the genetic associations with tracking in the 9th-grade partially reflects genetic variation in math skills that have been acquired prior to high school; however, the roles of attributes other than math ability, including the constellation of personality and motivational factors referred to as “non-cognitive” skills, are also likely important.^45^ We see potential hints of this here, as polygenic scores predict persistence in math *even* after controlling for the student’s math grades in the previous year. Other genetically influenced traits that are potentially influential for course placement are the student’s attention problems, behavioral and mental health difficulties, academic interests, motivations, and self-concept, and ability to elicit support and investment from adults.^46–48^

It is now well established that educational attainment is heritable^49^ and can be predicted from an individual’s DNA.^50–52^ What is less well-understood is *how* genetic differences between individuals lead to differences in educational outcomes. In order for genetics research to be of greater relevant to education research—and, ultimately, to education policy and practice—greater knowledge is needed about the developmental and social processes that connect students’ DNA to their educational outcomes.^53^ As sample sizes for GWAS continue to increase, more and more specific genetic variants associated with complex human phenotypes, like educational attainment, will continue to be identified. There are dangers associated with genetic research being used to justify an overly reductionistic or bio-deterministic account of educational outcomes.^54,55^ Here, however, we illustrate how DNA measures offer new opportunities for educational science. Specifically, we show that genetics can be used to identify leaks in the STEM pipeline and can refine our understanding who is benefitting (and who is not) from educational contexts.

## Methods

### Sample

The National Longitudinal Study of Adolescent to Adult Health (Add Health)^23^ is a nationally-representative cohort drawn from a probability sample of 80 U.S. high schools and 52 U.S. middle schools (in roughly 90 U.S. communities). Participating schools were representative of all U.S. schools in 1994–95 with respect to region, urban setting, school size, school type, and race or ethnic background. Add Health participants provided written informed consent for participation in all aspects of Add Health in accordance with the University of North Carolina School of Public Health Institutional Review Board guidelines that are based on the Code of Federal Regulations on the Protection of Human Subjects 45CFR46. This project was approved by the Stanford University IRB (eProtocol #: 35363).

We constructed our analytic sample as follows (see also Supplementary Table 1). At Wave 1, data was collected for *N* *=* 20,369 respondents. At Wave 3, respondents of the Add Health study, who were then 18–26 years old, were contacted and asked to give signed consent for the release of their official high school transcripts (AHAA).^25^ Transcripts were collected regardless of whether the student graduated from high school. At Wave 4, biospecimens were collected for genome-wide genotyping (described in^27,56^). Of those in the genetic sample, we focused on unrelated respondents of European ancestry, due to the problem of population stratification in diverse samples.^33,57^ Transcripts (*N* = 12,032) and genetic samples (*N* = 5,045 of European origin) were collected for partially overlapping subsets of the Wave 1 respondents. Our analytic sample therefore consisted of 3,635 European ancestry respondents with both genotypic and transcript data.

Descriptive statistics are contained in Supplementary Table 1. Compared to the full Add Health sample, our analytic sample had higher family SES, higher overall GPAs, and higher rates of post-secondary education. Missing information further reduced sample size in some analyses.

### Polygenic scores

Using results from the most recent educational attainment GWAS,^15^ we constructed a polygenic score using all single nucleotide polymorphisms (SNPs) with reported effect sizes that are also in the Add Health genetic dataset and where the effect allele can be reliably matched to the allele reported in the Add Health genetic data. We residualized the PGS on the top 10 principal components of genetic ancestry and then standardized the PGS based on the full set of respondents in the genetic dataset (*N* = 5045). A similar PGS has been used in previous work.^26,27^ Genotyped respondents who were not in the transcript dataset had a mean education-PGS of −0.11, whereas the genotyped respondents with transcript data had a mean education-PGS of 0.04 (Supplementary Table 1).

### Transcript coursetaking and course grades

Course content information obtained from the schools was used to identify the level of each course on a student’s transcript and to assign it a Classification of Secondary School Courses code. These codes were used to develop an ordinal indicator of the math course sequence, ranging from 0 (no math) to 9 (calculus). These indicators were developed by the AHAA project^25,58^ to be compatible with the 2000 National Assessment of Educational Progress High School Transcript Study^24^ and are based on population patterns of coursetaking as derived from the National Education Longitudinal Study of 1988.^59^ The percentages of students enrolled in each level at each year are in Supplementary Table 2. For analysis of 9th-grade coursetaking, math courses that focus on remedial skills (Basic/Remedial and General) were collapsed, as were math classes beyond Geometry (Algebra 2, Pre-Calculus, Advanced Math, and Calculus).

Students’ final math course grades at each year were obtained from transcripts and coded on a 0–4 scale (0 = *F*, 1 = *D*, 2 = *C*, 3 = *B*, 4 = *A*). If a student took the class pass/fail, withdrew, or received an incomplete, then his/her course grade is missing.^25^ A cumulative GPA was also computed from these transcript-based grades.

### Family and school SES

Family-of-origin SES was indexed using the first principal component of parental education, job status, income, and the number of benefits received (loadings were 0.58, 0.43, 0.49, and 0.49, respectively); see^27^ for additional information on this indicator. The family SES variable was standardized with respect to the full Wave I sample; the current analytic sample was more advantaged than the full sample (*M* = 0.34, SD = 1.16).

We used an indicator of school status used previously.^26,60^ Add Health administered an in-school survey to all students in participating high schools (*N* = 90,118). This information was used to calculate the percentage of students at each school who report that their mother graduated high school.

### Analyses

Our statistical models varied as a function of the outcome variable. For non-categorical outcome variables (e.g., number of advancing steps), we fit baseline generalized linear models of the form

1 $${{E}}\left( {{{y}}_{{{ij}}}} \right) = {{g}}^{ - 1}\left( {{{b}}_0 + {{b}}_1{\mathrm{PGS}}_{{{ij}}} + {\mathrm{controls}}} \right),$$

where *i* indexes school, *j* indexes individual, and an appropriate link function *g* is chosen given the distribution of the outcome *y*. For analyses of 9th-grade tracking, we fit ordered logistic regressions.^61^ For analysis of persistence, we used Poisson regressions. We fit interaction models of the form:

2 $${{E}}\left( {{{y}}_{{{ij}}}} \right) = {{g}}^{ - 1}\left( {{{b}}_0 + {{b}}_1{\mathrm{PGS}}_{{{ij}}} + {{b}}_2{\mathrm{School}}\,{\mathrm{Status}}_{{{ij}}} + {\mathrm{b}}_3{{PGS}}_{{\mathrm{ij}}} \cdot {\mathrm{School}}\,{\mathrm{Status}}_{{{ij}}} + {\mathrm{controls}}} \right).$$

For interaction models, we also included interactions between covariates and the key main effects, so as to guard against spurious findings from specification error.^62^ Thus, in models examining interactions between the education-PGS and School Status (as in Fig. 4), we also included interactions between PGS and sex, School Status and sex, PGS and birth year, and School Status and birth year (see Supplementary Table 3). Standard errors for key models (Tables S3–S6) are adjusted for school-based clustering using bootstrap resampling of the schools.^63^

For our ordinal categorical outcomes (e.g., tracking in 9th-grade), we consider cumulative link models.^61^ As used here, cumulative link models assert that:

3 $${\mathrm{Pr}}\left( {{{y}}_{{{ijk}}} \le {{j}}} \right) = {{f}}\left( {\theta _{{j}} - \left( {{{b}}_0 + {{b}}_1{\mathrm{PGS}}_{{{ij}}} + {\mathrm{controls}}} \right)} \right),$$

where *k* in [0,1,…, *K*] now indexes the category of the outcome *y*. We used a logit link, rendering this model equivalent to the proportional odds model.^64^ We again compute cluster-robust standard errors using school-based bootstrap resampling. One key assumption of this model is that the effect of the predictors does not vary across categories. We therefore also present results from alternative models (e.g., adjacent-category logit models) as robustness checks, see Supplement.

### Reporting summary

Further information on research design is available in the Nature Research Reporting Summary linked to this article.

## Supplementary information

Supplementary Information

Reporting Summary

## Data Availability

Data for this study come from the National Longitudinal Study of Adolescent to Adult Health (Add Health) and the Adolescent Health and Academic Achievement study (AHAA), which provided transcript data for Add Health participants. Restricted-use data files with phenotypic information from Add Health and AHAA can be obtained from the UNC Carolina Population Center following the procedures outlined in http://www.cpc.unc.edu/projects/addhealth/data/restricteduse. Genetic data on Add Health participants can be obtained from dbGaP at https://www.ncbi.nlm.nih.gov/projects/gap/cgi-bin/study.cgi?study_id=phs001367.v1.p1.

## Code availability

Code for analyses and figures is available in a GitHub repository at https://github.com/ben-domingue/transcripts-eaPGS.
