# Supplementary material for: Genetic associations with mathematics tracking and persistence in secondary school
Source: NPJ Sci Learn. 2020 Feb 5;5:1. doi: 10.1038/s41539-020-0060-2 (PMC7002519; doi:10.1038/s41539-020-0060-2)
Supplement: Supplementary file 1 — Supplementary Information [file 41539_2020_60_MOESM1_ESM.pdf]

Supplementary Information for

Genetic Associations with Mathematics Tracking and Persistence in Secondary School

K. Paige Harden, Benjamin W. Domingue, Daniel W. Belsky, Jason Boardman, Robert Crosnoe, Margherita Malanchini, Michel Nivard, Elliot M. Tucker-Drob, Kathleen Mullan Harris

Address correspondence to Paige Harden
Email: harden@utexas.edu
Phone: 512-471-1124

This PDF file includes:

Supplementary text
Supplementary Figures 1-2
Supplementary Tables 1-6
References for SI reference citations

Supplementary Information Text

Descriptives. Supplementary Tables 1 and 2, and Supplementary Figure 1 describe the analytic sample in more detail.

Supporting Tables. Supplementary Tables 3-6 contain estimates which we summarize in the main text.

Sensitivity Analyses. Supplementary Figure 2 consider non-parametric LOESS and adjacent category logit model (which do not require a homogeneous effect across categories; *c.f.*, Figure 4 from main text) as alternatives to the cumulative link models presented in main text.

Results from Family Fixed-Effects Models. We conducted exploratory analyses of a sample of $N = 441$ sibling pairs. (One participant per sibling pair was selected to be included in the primary analytic sample). Within-sibling differences in genotype are entirely random, and siblings raised in the same home share many aspects of their home environments. Testing whether siblings who differ in their education-PGS also differ in their subsequent education outcomes is, therefore, a strong test of genetic effects. We used family fixed-effects regression to compare siblings raised in the same nuclear family[1]. As with the school fixed-effects models, we fitted two lienar regression models that focused on deviations from the modal 9th-grade track (Algebra 1). The magnitude of the regression coefficients in the family fixed-effects models were consistent with other analyses in terms of sign (Table 3**)**: When comparing students with respect to taking at least Algebra 1 in the 9th-grade, students with higher education-PGS were less likely than their siblings to be placed in a remedial track (Pre-Algebra or lower) than in Algebra 1 ($b = 0.014$, $SE = 0.040$). Similarly, when examining students who were in Algebra 1 or higher in the 9th grade, students with higher education-PGS were more likely than their siblings to be placed in an advanced track (Geometry or higher) versus Algebra 1 ($b = 0.051$, $SE = 0.056$). However, neither effect was significantly different from zero.

To analyze persistence, we used the same Poisson regression analysis of advancing steps we used in other analyses. The magnitude of the regression coefficient for the education-PGS was attenuated relative to the coefficients estimated in similar models that do not involve a within-family comparison ($b = 0.049$, $SE = 0.043$) and not significantly different from zero (Table 4**)**.

Previous analyses of genetic data from trios of parents and offspring have shown that polygenic score associations with educational outcomes might partly reflect indirect genetic effects of the *parental* genotype, which are mediated via environments provided by the parent to all children in the family, *i.e.*, "genetic nurture" [2,3]. Consistent with this conclusion, the GWAS of educational attainment that was the basis of the education-PGS used in this study found that polygenic effect sizes were attenuated approximately 40% when comparing within-families relative to what was observed when comparing unrelated people [4]. As the effects of the education-PGS on tracking and persistence were not significantly different from zero, we cannot rule out the possibility that the education-PGS is reflecting the genetic endowment of the students' parents. This indirect genetic effect could operate through environmental advantages provided by the parents (such as parental knowledge and social capital) rather than a direct effect operating through the students' own skills and characteristics. The magnitude of the within-family coefficient for tracking, however, was similar to the effect estimated in between-family models, suggesting that these indirect genetic effects might be more relevant for understanding persistence than tracking.

Supplementary Figure 1. Construction of analytic sample.

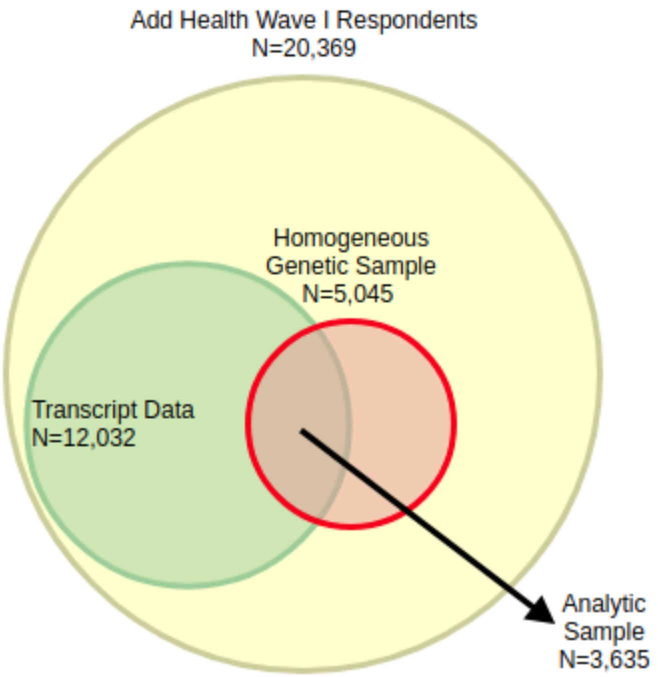

Supplementary Figure 2. Robustness analyses for math course placement in first year of postsecondary school. Each column considers the probability of placement in a math course relative to a course (or set of courses) immediately below as a function of EA PGS. The top row shows LOESS estimated probabilities while the bottom shows probabilities from a logit model (conditional on being female and of mean birthyear) for students in schools in the top and bottom half of the school SES distribution. Orange and blue represent relatively high and low status schools (respectively). The bottom panels represent fitted probabilities based on logistic regression models similar to the cumulative link models discussed in the main text.

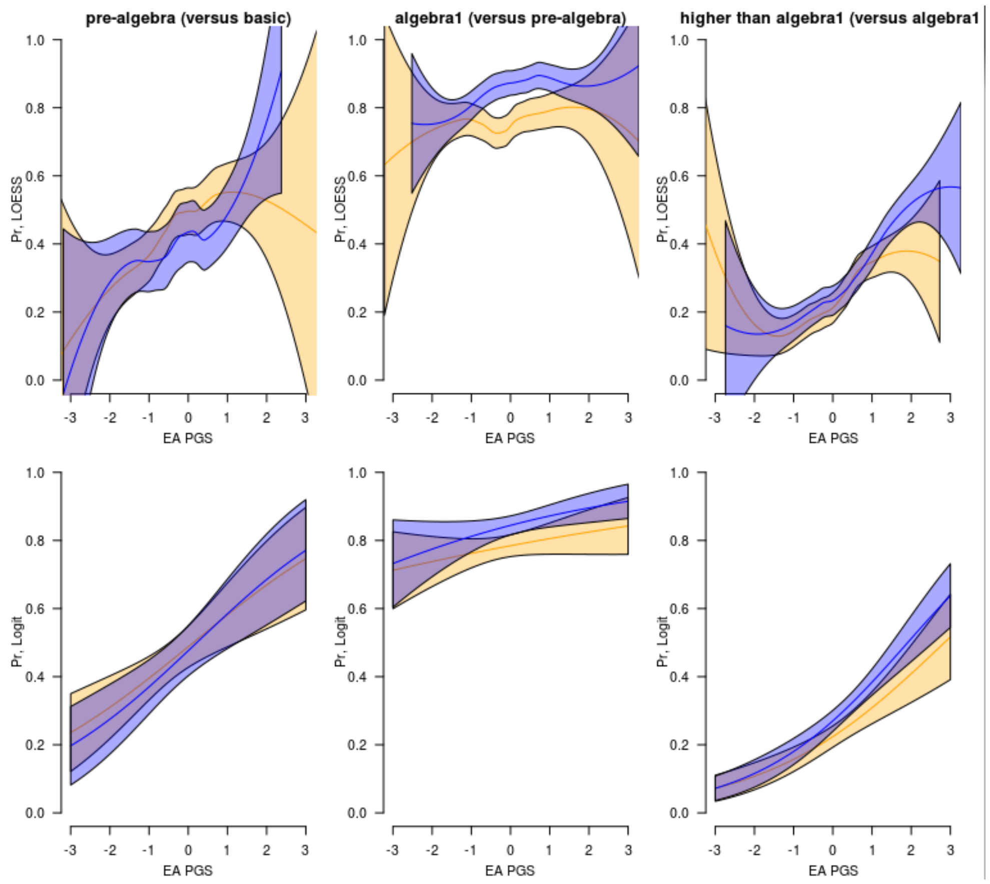

Supplementary Table 1. Comparison of analytic sample to full Add Health sample.

	All (*N*=20369, those with valid school id)			Transcript (*N*=12032)			Genetic (*N*=5045)			Analytic (*N*=3635)		
	Mean	*SD*	# NA	*Mean*	*SD*	# NA	*Mean*	*SD*	# NA	*Mean*	*SD*	# NA
Cumulative GPA	2.57	0.84	8408	2.57	0.84	71	2.69	0.81	1429	2.69	0.81	19
SES	0.01	1.35	1392	0.11	1.29	673	0.28	1.19	203	0.34	1.16	125
School Status	0.48	0.15	603	0.48	0.14	405	0.47	0.15	193	0.47	0.14	145
Female	0.5	0.5	2	0.53	0.5	0	0.53	0.5	0	0.54	0.5	0
Birthyear	78.83	1.73	15	78.91	1.73	8	78.97	1.74	1	79.02	1.75	1

Supplementary Table 2. Percentages of students in math courses during each year of high school.

	Year 1	Year 2	Year 3	Year 4
No math	2.6	5.2	17.6	44.7
Basic/Remedial	4.3	2.6	1.5	1.1
General	9.7	5.6	5.1	5.7
Pre-algebra	12.4	3.4	1.5	0.7
Algebra 1	51.3	21.4	6.6	2.3
Geometry	14.6	36.5	17.6	5.2
Algebra 2	4.2	19.1	24.7	8.3
Adv Math	0.3	2.7	4.8	4.8
Pre-calculus	0.4	3.1	19.2	17.2
Calculus	0.1	0.2	1.2	10.0

Supplementary Table 3. Regression coefficients from models of tracking.

A. Cumulative Link Models of starting math class in 9th grade.

A. Cumulative Link	Baseline	Plus SES	Plus School SES	Plus interactions
basic|pre-algebra	-1.829	-1.785	-1.464	-1.102
basic|pre-algebra.se	0.096	0.094	0.284	0.299
basic|pre-algebra.ci	-2.02, -1.62	-1.95, -1.6	-2.1, -0.979	-1.7, -0.539
pre-algebra|algebra1	-1.067	-0.987	-0.665	-0.330
pre-algebra|algebra1.se	0.090	0.082	0.279	0.295
pre-algebra|algebra1.ci	-1.24, -0.889	-1.14, -0.84	-1.28, -0.149	-0.953, 0.242
algebra1|higher than algebra1	1.457	1.665	1.994	2.246
algebra1|higher than algebra1.se	0.085	0.080	0.273	0.296
algebra1|higher than algebra1.ci	1.3, 1.63	1.52, 1.82	1.39, 2.5	1.65, 2.87
ea3	0.583	0.469	0.461	0.283
ea3.se	0.035	0.035	0.034	0.152
ea3.ci	0.516, 0.656	0.397, 0.53	0.398, 0.531	0.012, 0.588
sexmale	-0.261	-0.275	-0.275	-0.660
sexmale.se	0.061	0.060	0.063	0.238
sexmale.ci	-0.389, -0.143	-0.387, -0.147	-0.408, -0.163	-1.12, -0.184
birthyear	0.088	0.088	0.088	0.278
birthyear.se	0.035	0.034	0.033	0.087
birthyear.ci	0.0219, 0.156	0.0216, 0.152	0.0198, 0.147	0.13, 0.464
sespc.all		0.452	0.419	
sespc.all.se		0.039	0.039	
sespc.all.ci		0.382, 0.534	0.344, 0.5	
school.hsgrad			0.704	1.570
school.hsgrad.se			0.571	0.605
school.hsgrad.ci			-0.671, 1.74	0.344, 2.81
ea3:school.hsgrad				0.592
ea3:school.hsgrad.se				0.291
ea3:school.hsgrad.ci				-0.007, 1.11
ea3:sexmale				-0.049
ea3:sexmale.se				0.066
ea3:sexmale.ci				-0.176, 0.0783
ea3:birthyear				-0.006
ea3:birthyear.se				0.020
ea3:birthyear.ci				-0.0431, 0.0363
school.hsgrad:sexmale				0.833
school.hsgrad:sexmale.se				0.515
school.hsgrad:sexmale.ci				-0.189, 1.83
school.hsgrad:birthyear				-0.408
school.hsgrad:birthyear.se				0.174
school.hsgrad:birthyear.ci				-0.763, -0.106
N	3367	3367	3367	3367

B. Logistic regression models predicting a student starting math in 9th grade in Algebra 1 or above (note: family-based analyses use linear probability models).

B. Algebra 1 or above	Baseline	Plus SES	School-based	Family-based
ea3	0.388	0.299	0.387	0.014
ea3.se	0.052	0.051	0.054	0.040
ea3.ci	0.29, 0.499	0.204, 0.405	0.294, 0.504	-0.058, 0.107
sexmale	-0.191	-0.208	-0.245	-0.169
sexmale.se	0.079	0.079	0.086	0.073
sexmale.ci	-0.36, -0.051	-0.352, -0.0486	-0.43, -0.1	-0.308, -0.0283
birthyear	0.072	0.075	0.174	0.020
birthyear.se	0.051	0.052	0.078	0.030
birthyear.ci	-0.0229, 0.174	-0.0198, 0.174	0.0378, 0.324	-0.0479, 0.0717
sespc.all		0.380		
sespc.all.se		0.050		
sespc.all.ci		0.286, 0.482		
(Intercept)	0.728	0.680	17.615	0.952
(Intercept).se	0.098	0.107	8.180	0.135
(Intercept).ci	0.561, 0.939	0.483, 0.916	0.0914, 17.8	0.538, 1.08
N	2719	2719	2719	332

C. Logistic regression models predicting a student starting math in 9th grade above Algebra 1 (note: family-based analyses use linear probability models).

C. Above algebra 1	Baseline	Plus SES	School-based	Family-based
ea3	0.534	0.451	0.587	0.051
ea3.se	0.047	0.044	0.047	0.056
ea3.ci	0.442, 0.629	0.361, 0.54	0.501, 0.681	-0.0709, 0.156
sexmale	-0.212	-0.220	-0.199	0.007
sexmale.se	0.089	0.096	0.092	0.051
sexmale.ci	-0.401, -0.0519	-0.399, -0.0392	-0.384, -0.0282	-0.0736, 0.125
birthyear	0.079	0.076	0.106	-0.011
birthyear.se	0.040	0.038	0.045	0.024
birthyear.ci	0.00596, 0.151	-0.00188, 0.15	0.0235, 0.196	-0.041, 0.0469
sespc.all		0.379		
sespc.all.se		0.075		
sespc.all.ci		0.236, 0.522		
(Intercept)	-1.081	-1.292	-17.642	-0.042
(Intercept).se	0.099	0.108	8.085	0.351
(Intercept).ci	-1.27, -0.879	-1.53, -1.09	-18.7, -0.533	-0.247, 0.99
N	2400	2400	2400	334

Supplementary Table 4. Regression coefficients from models of persistence.

	baseline	baseline plus ses.both	baseline plus ses.both+math9	baseline.interaction	baseline.interaction.math9control	school	fam
(Intercept)	0.541	0.383	0.466	0.255	0.313	0.533	0.928
(Intercept).se	0.024	0.089	0.088	0.082	0.088	0.164	0.337
(Intercept).ci	0.494, 0.589	0.209, 0.549	0.292, 0.623	0.0755, 0.396	0.126, 0.477	-0.00503, 0.55	0.0163, 1.3
ea3	0.139	0.096	0.087	0.268	0.232	0.117	0.049
ea3.se	0.011	0.013	0.012	0.043	0.040	0.013	0.043
ea3.ci	0.117, 0.159	0.072, 0.12	0.061, 0.11	0.183, 0.352	0.143, 0.304	0.091, 0.138	-0.038, 0.134
sexmale	-0.058	-0.058	-0.050	-0.126	-0.101	-0.060	-0.003
sexmale.se	0.019	0.019	0.019	0.069	0.064	0.019	0.076
sexmale.ci	-0.0975, -0.0229	-0.0937, -0.0205	-0.0885, -0.0146	-0.248, 0.0224	-0.211, 0.0415	-0.0992, -0.0269	-0.199, 0.106
birthyear	-0.008	-0.009	-0.012	0.005	-0.009	0.002	-0.044
birthyear.se	0.010	0.010	0.011	0.036	0.038	0.012	0.022
birthyear.ci	-0.0262, 0.0119	-0.0276, 0.011	-0.0342, 0.0113	-0.0584, 0.0819	-0.0839, 0.0635	-0.0188, 0.0259	-0.0808, 0.00843
sespc.all		0.120	0.106				
sespc.all.se		0.015	0.014				
sespc.all.ci		0.089, 0.147	0.0779, 0.133				
school.hsgrad		0.234	0.210	0.613	0.633		
school.hsgrad.se		0.171	0.166	0.154	0.153		
school.hsgrad.ci		-0.097, 0.572	-0.099, 0.534	0.349, 0.973	0.341, 0.943		
math.start-1			-0.221		-0.112		
math.start-1.se			0.038		0.121		
math.start-1.ci			-0.293, -0.147		-0.339, 0.142		
math.start1			-0.059		0.068		
math.start1.se			0.044		0.183		
math.start1.ci			-0.147, 0.020		-0.285, 0.429		
ea3:school.hsgrad				-0.304	-0.282		
ea3:school.hsgrad.se				0.074	0.072		
ea3:school.hsgrad.ci				-0.443, -0.147	-0.41, -0.131		

ea3:sexmale				0.008	0.010		
ea3:sexmale.se				0.022	0.020		
ea3:sexmale.ci				-0.0308, 0.0543	-0.0279, 0.0496		
ea3:birthyear				-0.001	0.000		
ea3:birthyear.se				0.005	0.006		
ea3:birthyear.ci				-0.0098, 0.00947	-0.00937, 0.0118		
school.hsgrad:sexmale				0.135	0.106		
school.hsgrad:sexmale.se				0.139	0.128		
school.hsgrad:sexmale.ci				-0.164, 0.381	-0.185, 0.323		
school.hsgrad:birthyear				-0.027	-0.007		
school.hsgrad:birthyear.se				0.064	0.066		
school.hsgrad:birthyear.ci				-0.172, 0.0868	-0.141, 0.115		
ea3:math.start-1					0.026		
ea3:math.start-1.se					0.023		
ea3:math.start-1.ci					-0.0182, 0.0703		
ea3:math.start1					-0.003		
ea3:math.start1.se					0.029		
ea3:math.start1.ci					-0.055, 0.0543		
school.hsgrad:math.start-1					-0.307		
school.hsgrad:math.start-1.se					0.236		
school.hsgrad:math.start-1.ci					-0.791, 0.133		
school.hsgrad:math.start1					-0.171		
school.hsgrad:math.start1.se					0.334		
school.hsgrad:math.start1.ci					-0.891, 0.472		
N	3367	3367	3367	3367	3367	3367	441

Supplementary Table 5. Regression coefficients from year-by-year models of persistence.

	Grade 9 to Grade 10		Grade 10 to Grade 11		Grade 11 to Grade 12	
(Intercept)	1.930	1.219	0.988	0.296	0.054	-0.507
(Intercept).se	0.466	0.461	0.293	0.319	0.246	0.264
(Intercept).ci	0.976, 2.86	0.375, 2.23	0.468, 1.61	-0.296, 0.92	-0.394, 0.563	-1.01, 0.0303
ea3	0.288	0.234	0.220	0.143	0.167	0.123
ea3.se	0.094	0.102	0.046	0.052	0.044	0.038
ea3.ci	0.129, 0.499	0.045, 0.444	0.127, 0.307	0.043, 0.237	0.078, 0.249	0.045, 0.199
sespc.all	0.199	0.104	0.233	0.182	0.154	0.109
sespc.all.se	0.059	0.069	0.060	0.059	0.044	0.043
sespc.all.ci	0.082, 0.308	-0.0336, 0.246	0.111, 0.352	0.068, 0.29	0.0569, 0.232	0.0219, 0.196
school.hsgrad	2.931	2.717	1.650	1.654	0.652	0.720
school.hsgrad.se	0.891	0.945	0.612	0.688	0.508	0.498
school.hsgrad.ci	1.36, 4.61	1.02, 4.59	0.394, 2.84	0.336, 3	-0.393, 1.6	-0.266, 1.72
sexmale	-0.302	-0.183	-0.236	-0.117	0.161	0.245
sexmale.se	0.178	0.168	0.109	0.103	0.093	0.094
sexmale.ci	-0.657, 0.0389	-0.491, 0.167	-0.476, -0.0458	-0.337, 0.0591	-0.00457, 0.363	0.0739, 0.415
birthyear	-0.050	-0.062	0.021	0.017	-0.023	-0.033
birthyear.se	0.056	0.058	0.036	0.039	0.026	0.026
birthyear.ci	-0.17, 0.0637	-0.183, 0.0453	-0.0478, 0.0877	-0.0666, 0.0938	-0.0737, 0.0254	-0.0797, 0.0188
gpa		0.370		0.302		0.231
gpa.se		0.080		0.046		0.054
gpa.ci		0.218, 0.527		0.222, 0.389		0.125, 0.326
N	3676	3652	3573	3539	3087	3050

Supplementary Table 6. Regression coefficients from models of highest math credential.

	Highest math is at least Algebra 2	Highest math is calculus
(Intercept)	-0.041	-3.534
(Intercept).se	0.331	0.712
(Intercept).ci	-0.644, 0.641	-4.8, -1.99
ea3	0.466	0.897
ea3.se	0.158	0.246
ea3.ci	0.167, 0.781	0.414, 1.37
school.hsgrad	1.867	2.406
school.hsgrad.se	0.647	1.456
school.hsgrad.ci	0.551, 3.1	-0.978, 4.67
sexmale	-0.350	-0.662
sexmale.se	0.300	0.573
sexmale.ci	-0.958, 0.19	-1.72, 0.476
birthyear	0.232	0.367
birthyear.se	0.092	0.158
birthyear.ci	0.0667, 0.421	0.0404, 0.662
ea3:school.hsgrad	0.313	-0.385
ea3:school.hsgrad.se	0.314	0.478
ea3:school.hsgrad.ci	-0.296, 0.9	-1.18, 0.592
ea3:sexmale	-0.029	0.015
ea3:sexmale.se	0.081	0.137
ea3:sexmale.ci	-0.178, 0.14	-0.247, 0.274
ea3:birthyear	0.031	-0.014
ea3:birthyear.se	0.027	0.038
ea3:birthyear.ci	-0.0226, 0.0824	-0.0972, 0.0549
school.hsgrad:sexmale	-0.010	1.148
school.hsgrad:sexmale.se	0.672	1.165
school.hsgrad:sexmale.ci	-1.22, 1.28	-1.16, 3.48
school.hsgrad:birthyear	-0.454	-0.550
school.hsgrad:birthyear.se	0.200	0.297
school.hsgrad:birthyear.ci	-0.857, -0.0753	-1.05, 0.119
N	3367	3367

Supplementary References

1. Fletcher, J. M. Social interactions and college enrollment: A combined school fixed effects/instrumental variables approach. *Soc Sci Res* **52**, 494–507 (2015).
2. Kong, A. *et al.* The nature of nurture: Effects of parental genotypes. *Science* **359**, 424–428 (2018).
3. Koellinger, P. D. & Harden, K. P. Using nature to understand nurture. *Science* **359**, 386–387 (2018).
4. Lee, J. J. *et al.* Gene discovery and polygenic prediction from a genome-wide association study of educational attainment in 1.1 million individuals. *Nature Genetics* **50**, 1112–1121 (2018).
